# Micro-Environmental Mechanical Stress Controls Tumor Spheroid Size and Morphology by Suppressing Proliferation and Inducing Apoptosis in Cancer Cells

**DOI:** 10.1371/journal.pone.0004632

**Published:** 2009-02-27

**Authors:** Gang Cheng, Janet Tse, Rakesh K. Jain, Lance L. Munn

**Affiliations:** 1 Edwin L. Steele Laboratory of Tumor Biology, Department of Radiation Oncology, Massachusetts General Hospital and Harvard Medical School, Boston, Massachusetts, United States of America; 2 Department of Chemical Engineering, Massachusetts Institute of Technology, Cambridge, Massachusetts, United States of America; Ordway Research Institute, United States of America

## Abstract

**Background:**

Compressive mechanical stress produced during growth in a confining matrix limits the size of tumor spheroids, but little is known about the dynamics of stress accumulation, how the stress affects cancer cell phenotype, or the molecular pathways involved.

**Methodology/Principal Findings:**

We co-embedded single cancer cells with fluorescent micro-beads in agarose gels and, using confocal microscopy, recorded the 3D distribution of micro-beads surrounding growing spheroids. The change in micro-bead density was then converted to strain in the gel, from which we estimated the spatial distribution of compressive stress around the spheroids. We found a strong correlation between the peri-spheroid solid stress distribution and spheroid shape, a result of the suppression of cell proliferation and induction of apoptotic cell death in regions of high mechanical stress. By compressing spheroids consisting of cancer cells overexpressing anti-apoptotic genes, we demonstrate that mechanical stress-induced apoptosis occurs via the mitochondrial pathway.

**Conclusions/Significance:**

Our results provide detailed, quantitative insight into the role of micro-environmental mechanical stress in tumor spheroid growth dynamics, and suggest how tumors grow in confined locations where the level of solid stress becomes high. An important implication is that apoptosis via the mitochondrial pathway, induced by compressive stress, may be involved in tumor dormancy, in which tumor growth is held in check by a balance of apoptosis and proliferation.

## Introduction

The growth of solid tumors is strongly influenced by its microenvironment. Besides well-studied microenvironmental parameters, such as hypoxia [1], [2] and angiogenesis [3]–[5], mechanical stresses also play an important role. For a solid tumor to grow in a confined space defined by the surrounding tissue, it must overcome the resulting compressive forces. It has been shown that tumors and their associated stroma are mechanically stiffer than the corresponding normal host tissue [6], and that mechanical compression in such an environment can collapse blood and lymphatic vessels [7]. However, our understanding of how this compression directly influences tumor growth is limited. Various hypotheses have been proposed regarding the involvement of mechanical stresses in tumor development [8]–[11], and Helmlinger et al. [12] conducted the first quantification of spheroid growth inhibition in agarose gels. They found that human colon carcinoma spheroids can grow to a maximum size of 400 µm (diameter) in 0.5% (w/v) agarose, but only 50 µm in 1.0% agarose (which is less compliant). This was associated with an increase in cell packing and a decrease in cell proliferation. They also showed that such inhibition of tumor growth can be reversed by releasing the spheroids from the gel. Yet several key questions remain unanswered, including: (1) What is the nature of the stress field around growing tumor spheroids? (2) Can local solid stress distribution affect the shape of tumor spheroids? (3) Does solid stress distribution also affect cell phenotype in different regions of individual spheroids? (4) What is the intracellular pathway that regulates the solid stress-induced phenotypic change(s)? These questions are critical for a fundamental understanding of solid tumor growth dynamics.

In this study, we show that the accumulating solid stress in agarose gels around growing tumor spheroids (non-metastatic murine mammary carcinoma 67NR cells unless stated otherwise) can be measured using co-embedded fluorescent micro-beads (diameter = 1 µm) as markers for strain in the gel: agarose gels are resistant to degradation by cancer cell proteinases [13], and thus allow studies of solid stress accumulation independent of cell invasion. We demonstrate that the shape of the solid stress field dictates the shape of tumor spheroids and that this effect is due to suppression of cell proliferation and induction of cell apoptosis in regions of high solid stress. Finally, we elucidate the molecular mechanism for the solid stress-induced apoptosis.

## Results

### Growing tumor spheroids progressively compress the surrounding matrix

**Figure 1:**
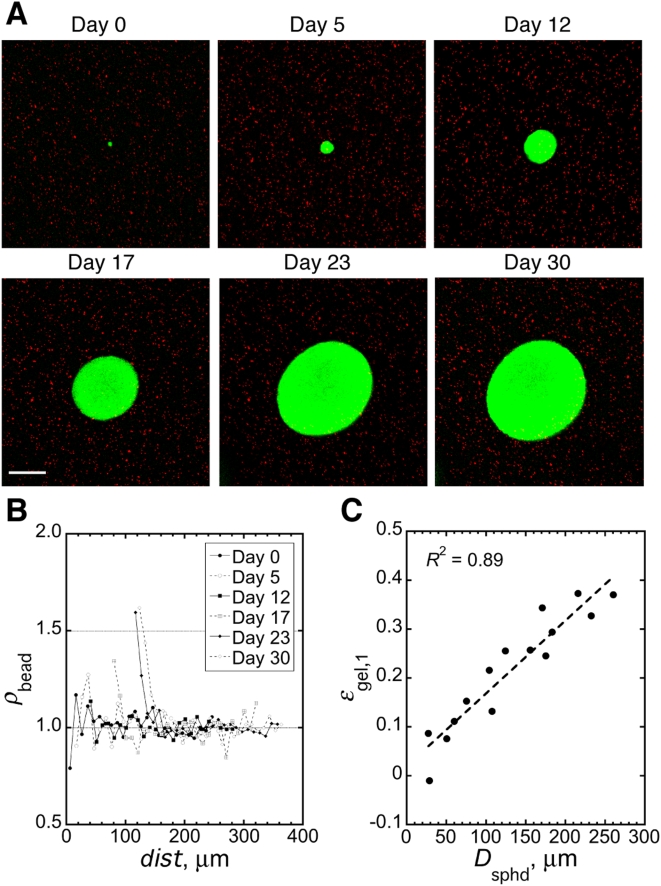
Mechanical stress accumulates around growing tumor spheroids. (*A*) A growing spheroid (green) and its surrounding micro-beads (red). Scale bar = 100 µm. (*B*) Quantification of relative micro-bead density (*ρ*_bead_) in 10-µm thick shells of agarose gel around the growing spheroid shown in *A* as a function of the distance of the shell from spheroid center (*dist*). (*C*) Correlation between spheroid diameter (*D_sphd_*) and the strain in the first 10-µm thick shell of agarose gel (*ε*_gel,1_) around spheroids. *R* is the linear regression coefficient; slope of the regression line is significantly greater than zero (*p*<0.0001).

Fig. 1*A* shows the growth of a typical tumor spheroid (green) co-embedded with micro-beads (red) in 0.5% agarose gel for 30 days (see experimental setup in Supplementary Fig. S1*A*). Analysis via 3D confocal microscopy revealed that the agarose gel was progressively compressed by the growing spheroid (Fig. 1*B*). At early time points, there was much fluctuation in the micro-bead density (*ρ*_beads_), mainly because the spheroid was still fairly small and, as a result, the sampling volume for measuring *ρ*_beads_ was limited. Significant increases in *ρ*_beads_ started to appear at day 17 when the spheroid diameter (*D*_sphd_) was only ∼150 µm. By day 30, *D*_sphd_ had reached ∼250 µm and *ρ*_beads_ in the first 10 µm-thick shell of agarose gel (*ρ*_beads,1_) was ∼1.6 times that of unstressed gels, corresponding to a 37.5% strain (*ε*_gel,1_). Significant strain was limited to the immediate vicinity of the spheroid: *ρ*_beads_ decreased to its control level within ∼50 µm from the spheroid surface (Fig. 1*B*, day 30 curve). The relationship between spheroid growth and the resulting strain in the agrose gel is linear in the range of *D*_sphd_ measured in our study (Fig. 1*C*). From published mechanical properties of 0.5% agarose gel [14], we estimated that the spheroid in Fig. 1*A* imposed ∼28 mmHg of solid stress on the immediately adjacent matrix at day 30, similar to the value estimated theoretically by Helmlinger et al. [12]. For spheroids grown in 1.0% gel, the sampling volume for the quantification of micro-bead density was too small because most spheroids could not grow larger than 50 µm in diameter.

### Tumor spheroid shape correlates strongly with the shape of local solid stress field

**Figure 2:**
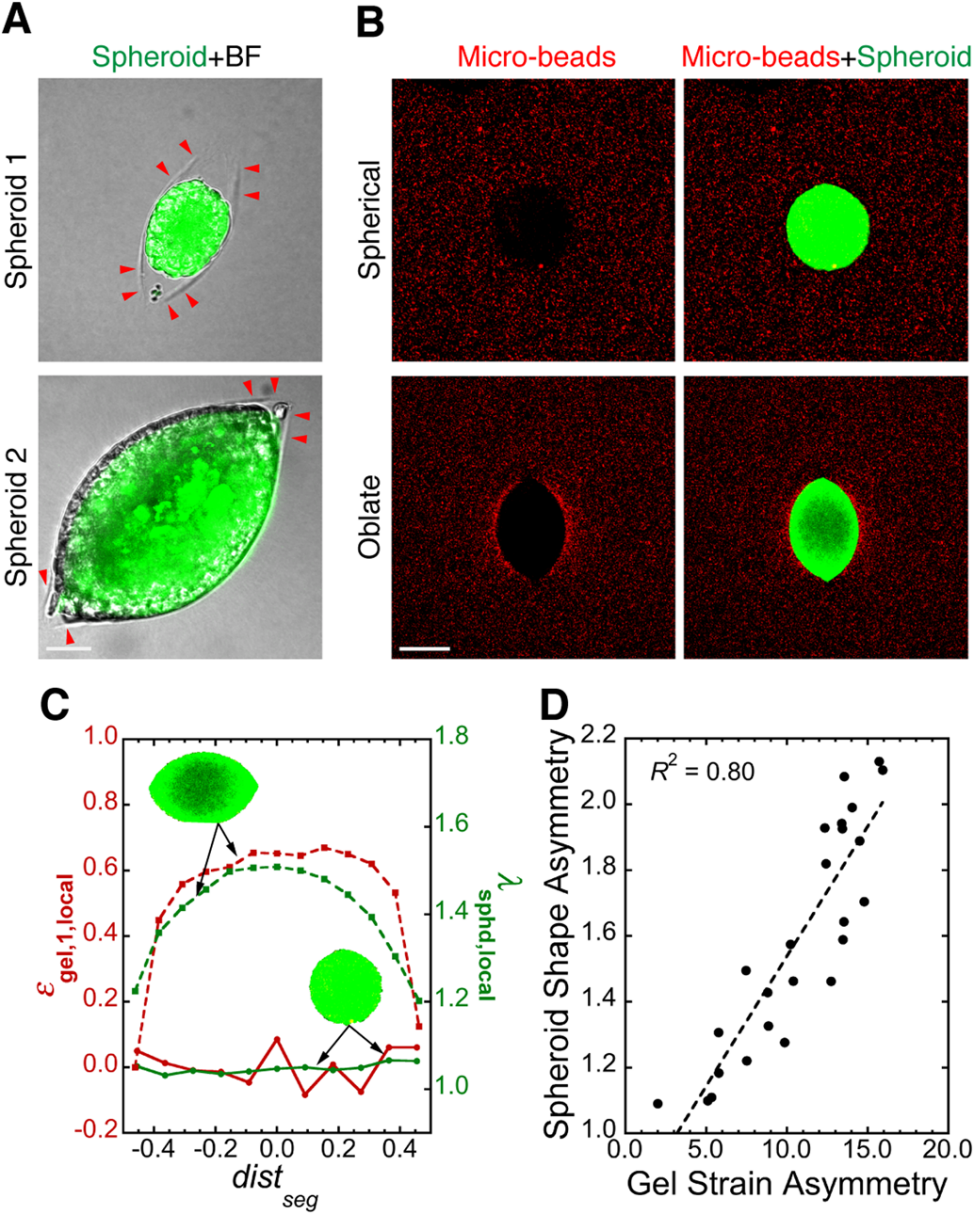
Mechanical stress distribution controls tumor spheroid shape. (*A*) Agarose gel can fail under tension from growing tumor spheroids (green). Red arrowheads indicate the edge of planar cracks in the agarose gel (BF: bright-field image taken in Nomarski mode). Scale bar = 50 µm. (*B*) Spheroids (green) of different shapes and their surrounding stress fields visualized by micro-beads (red). Scale bar = 150 µm. (*C*) Relationship between local strain in agarose gel (*ε*_gel,1,local_) and local spheroid deformation (*λ*_sphd,local_) for the spheroids (green, inset) shown in *A*. *dist*_seg_ is the distance of spheroid segments from spheroid center normalized over the length of the major axis. (*D*) Correlation between the asymmetry in spheroid shape and in the corresponding strain in the surrounding agarose gel, showing that spheroids are more deformed along the direction of higher stress. Each data point is for one spheroid. *R* is the linear regression coefficient; slope of the regression line is significantly greater than zero (*p*<0.0001). Methods for image analysis in *C* and *D* are described in Supplementary Methods S1, Supplementary Fig. S2, Movie S2 and Movie S3.

In the vicinity of some spheroids, the gel failed under the tension produced by the spheroid growth. This resulted in micro-scale planar cracks in the gel (Fig. 2*A*, arrowheads). Although the spheroids selected for analysis in Fig. 1 did not reside within such cracks and were all nearly spherical in shape, spheroids that did exist within cracks adopted oblate shapes (i.e., flattened spheres, Fig. 2*A*; also see Movie S1 for 3D rendering). Quantification of micro-bead density around typical spherical and oblate spheroids revealed a strong correlation between spheroid shape and the shape of the local mechanical stress field (Fig. 2*B, 2C*). This was further confirmed by the results from similar analysis on ∼25 spheroids of various shapes (Figs. 2*D*). Helmlinger et al. observed a similar phenomenon by growing spheroids in 1-mm capillary glass tubes: the spheroids elongated along the tube axis, presumably in response to the local stress field [12].

### High solid stress suppresses cell proliferation and induces apoptotic cell death in tumor spheroids

**Figure 3:**
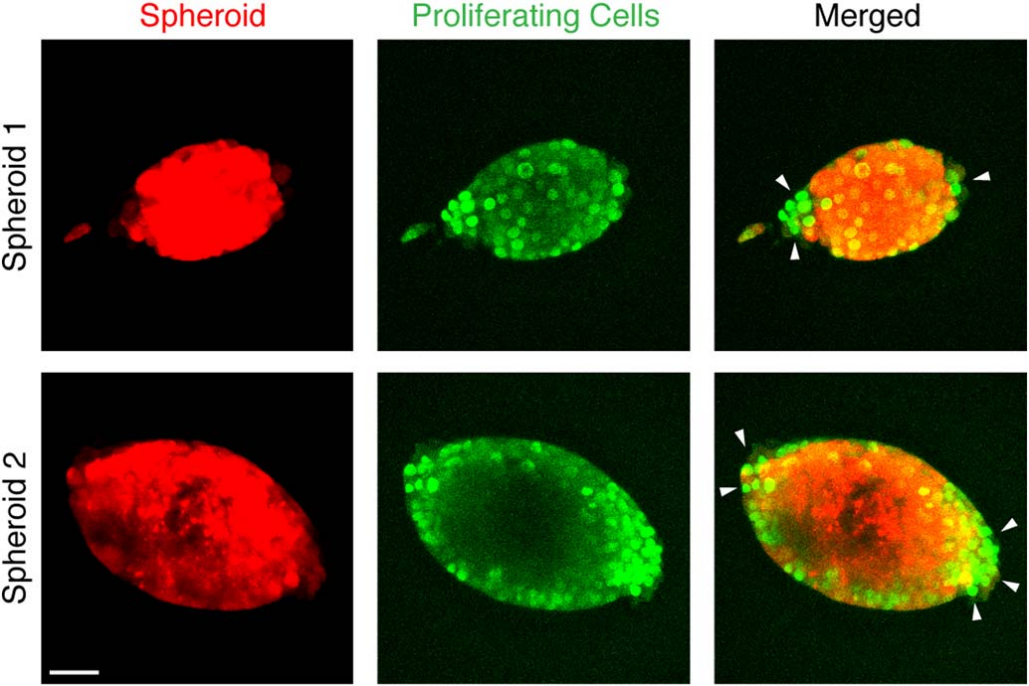
Cancer cell proliferation (green) in tumor spheroids (red) is suppressed in the direction of higher mechanical stress (i.e., in the direction of the minor axis of oblate spheroids). Arrowheads indicate the regions with more cell proliferation. Scale bar = 50 µm.

**Figure 4:**
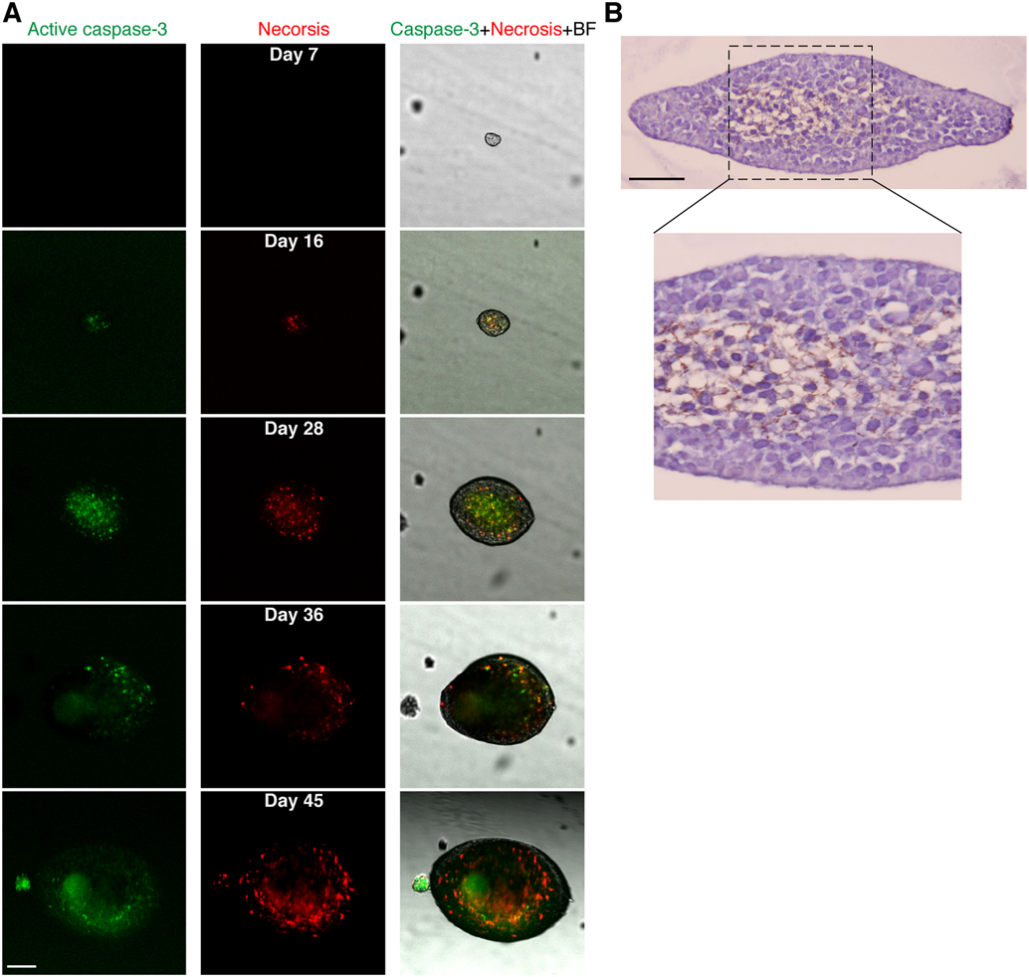
Mechanical stress induces apoptotic cell death in tumor spheroids. (*A*) The development of apoptosis (green) and secondary necrosis (red) in a growing spheroid embedded in 0.5% agarose gel. Scale bar = 100 µm. (*B*) Immunohistochemical staining (TUNEL and hematoxylin) confirming the results in *A*. Image in the lower panel shows detail of the area within the dashed line in the image in the upper panel. Scale bar = 50 µm.

**Figure 5:**
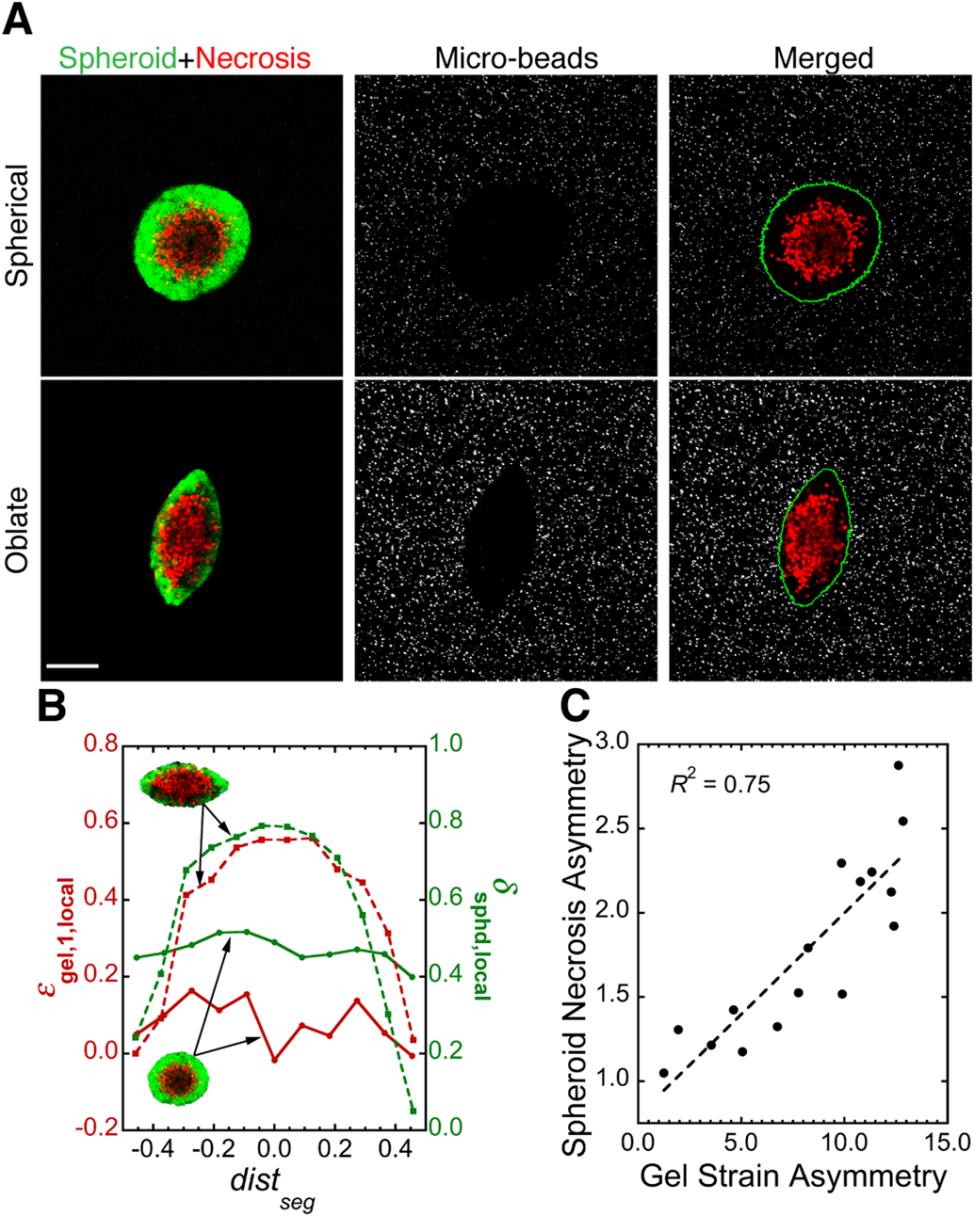
Mechanical stress distribution correlates strongly with the distribution of cell death in tumor spheroids. (*A*) Live spheroids (green) of different shapes, their internal cell death (red), and their surrounding stress fields visualized by micro-beads (gray). The green line in the right column shows the edge of spheroids. Scale bar = 100 µm. (*B*) Relationship between local strain in agarose gel (*ε*_gel,1,local_) and local necrotic fraction in spheroids (*δ*_sphd,local_) for the two spheroids shown in *A*. (*C*) Correlation between the asymmetry in spheroid necrotic fraction and in the corresponding strain in the surrounding agarose gel, showing that there is more cell death along the direction of higher compressive stress. Each data point is for one spheroid. *R* is the linear regression coefficient; slope of the regression line is significantly greater than zero (*p*<0.0001). Methods for image analysis in *B* and *C* are described in Supplementary Methods, Supplementary Fig. S2, Movie S3 and Movie S4.

To investigate potential phenotypic changes that solid stress induces in cancer cells, we evaluated cell proliferation and apoptosis in the spheroids. Cell division near regions of higher stress (i.e., in the direction of the minor axis of oblate spheroids) was reduced compared to regions of lower stress (Fig. 3). To check how compressive stress affects cell viability, we examined apoptosis and necrosis in live spheroids cultured in 0.5% (Fig. 4) or 1.0% agarose gels (Supplementary Fig. S3). In the 0.5% gel, apoptosis and necrosis appeared when the spheroids were only ∼50 µm in diameter (Fig. 4*A*, day 12) and continued to increase, becoming extensive by day 28. Subsequently, the apoptotic areas were gradually replaced by necrosis until, by day 45, necrosis had almost reached the surface of the spheroid. In 1.0% agarose gel, the spheroid could not grow larger than ∼30 µm in diameter, and apoptosis and necrosis were detected even in these small spheroids (Supplementary Fig. S3). These observations in live spheroids were confirmed by immunohistochemical staining of fixed samples (Fig. 4*B*). Furthermore, there was a strong correlation between the local fraction of cell death in spheroids and the local strain in the agarose gel (Fig. 5).

**Figure 6:**
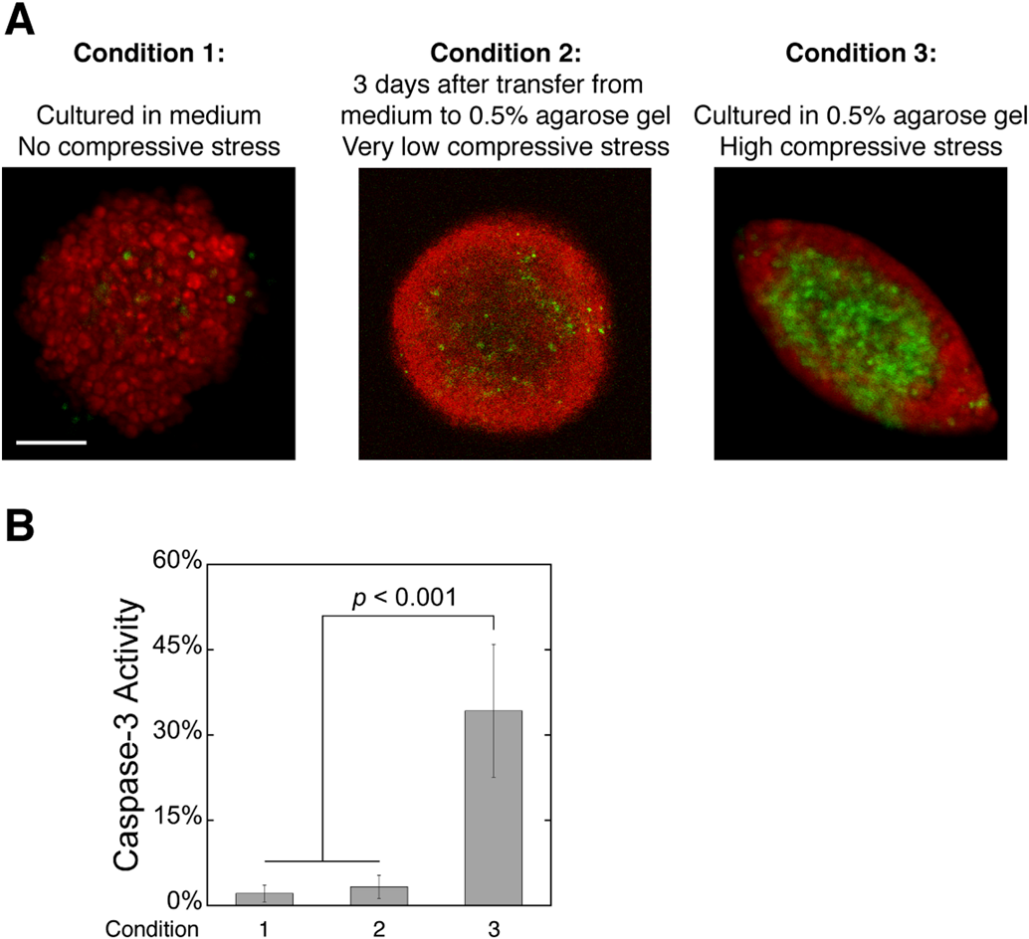
Agarose gel toxicity or limitations of nutrients, growth factors and oxygen is not responsible for the apoptosis observed in spheroids under high levels of compressive stress. (*A*) Caspase-3 activity (green) in tumor spheroids (red) cultured in free suspension (Condition 1), transferred to 0.5% agarose gel for 3 days after reaching plateau-phase in free suspension (Condition 2), or cultured from single cells in 0.5% agarose gel (Condition 3). Scale bar = 100 µm. (*B*) Quantification of the fraction of apoptotic cells in tumor spheroids cultured under the 3 conditions in *A*.

To verify that limitations of nutrients, growth factors or oxygen were not responsible for the apoptotic cell death in Fig. 4, we assessed apoptosis in spheroids grown to similar sizes in free suspension (hanging droplets). The free suspension cultures had no external confining matrix and little apoptosis (Fig. 6*A*, Condition 1). To check whether cell-agarose interactions contributed to cell death, we transferred free suspension spheroids into 0.5% agarose gels where they were allowed to acclimate for 3 days. As opposed to spheroids grown from single cells in agarose gel (Fig. 6*A*, Condition 3), the transferred spheroids did not have time to accumulate significant levels of solid stress, and they had much less cell death (Fig. 6*A*, Condition 2). Thus, it is unlikely that gel toxicity or limitations of nutrients, growth factors or oxygen were responsible for the apoptosis observed in the stressed spheroids.

### Externally-applied compression mimics growth-induced stress

**Figure 7:**
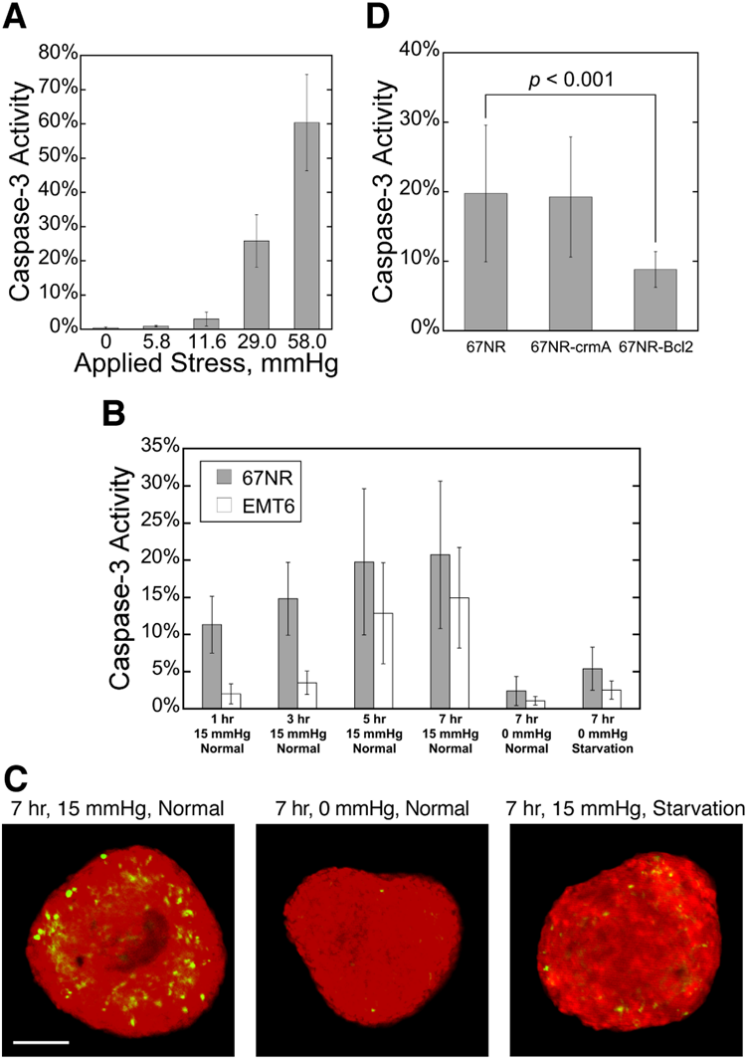
Mechanical stress-induced cell death in tumor spheroids acts via the mitochondrial pathway. (*A*) Caspase-3 activity increases in monolayers of cancer cells in response to higher external stress. Cells were compressed for 17 hr. (*B*) Caspase-3 activity in spheroids made from two different cancer cell lines in response to different external stress (0 mmHg or 15 mmHg) and nutrient conditions (Normal: normal medium; Starvation: no glucose, no serum and 1% oxygen). (*C*) Typical caspase-3 activity (green) in spheroids (red) cultured in 3 of the conditions in *B*. Scale bar = 100 um. (*D*) Bcl-2 over-expression inhibits stress-induced apoptosis, but crmA transduction does not. Spheroids were compressed with 15 mmHg for 7 hr while being supplied with normal medium.

If compressive stress causes apoptotic cell death in tumor spheroids, it should not matter whether it is growth-induced or externally-applied. Therefore, to quantify the effect of solid stress on cell apoptosis under controlled conditions, we first compressed monolayers of cancer cells for 17 hr with pressures ranging from 0 mmHg to 60 mmHg (see experimental setup in Supplementary Fig. S1*B*) and observed increased apoptosis with increased stress level (Fig. 7*A*). We then transferred spheroids approximately 300 µm in diameter from free suspension into 1% agarose gel and cultured them under three conditions: normal medium without external compression, starvation medium (no glucose, no serum and 1% oxygen) without compression or normal medium with external compression (see experimental setup in Supplementary Fig. S1*C*). Caspase-3 activity was evaluated at 1 hr, 3 hr, 5 hr and 7 hr (Fig. 7*B, 7C*; the relatively short compression times were chosen to minimize potential complication of nutrient/growth factor/oxygen limitations in the 3D culture). Compression dramatically increased caspase-3 activity, which leveled off after 5 hr. Spheroids that were starved but un-stressed had much less apoptosis than those in the compressed samples, again indicating that limitations of glucose, serum and oxygen alone do not account for the levels of cell death demonstrated in Fig. 4.

### The mitochondrial pathway regulates mechanical stress-induced cell apoptosis in tumor spheroids

Finally, we investigated which of the two major apoptotic pathways [15] regulates the solid stress-induced cell apoptosis. We transduced cancer cells to overexpress crmA which inhibits initiator caspases including caspase-1 and caspase-8 in the death-receptor pathway [16], or Bcl-2, a well-known inhibitor of multiple caspases in the mitochondrial pathway [17]. Free suspension spheroids of wild-type or transduced cells grown to ∼300 µm in diameter were transferred into 1% agrose gel and compressed as described before. Fig. 7*D* shows that Bcl-2 overexpression significantly reduces compression-induced cell death in the spheroids while crmA over-expression has little effect. Similarly, overexpression of crmA in a highly metastatic murine breast carcinoma cell line EMT6, which has a high level of endogenous Bcl-2 expression [18], did not further protect from compression-induced caspase-3 activity (data not shown), suggesting that the mitochondrial pathway regulates solid stress-induced apoptosis.

## Discussion

The present study addressed several remaining questions concerning the effect of compressive stress on the growth dynamics of solid tumors. Although empirical mathematical models such as the well-known Gompertzian growth curve [19] and the more recent “universal growth law” [20]–[22] can predict the enlargement of many solid tumors with good accuracy, they do not explicitly consider cell dynamics inside the tumors. In particular, the invariable emergence of a plateau phase after tumors have reached a certain size has never been satisfactorily explained. As most solid tumors larger than 1 mm in diameter induce angiogenesis [3], nutrient or oxygen depletion should not limit tumor growth. Our study shows that growth-induced solid stress can affect cell phenotype, and suggests that there may be a “dynamic equilibrium” of proliferation and apoptosis that maintains tumor size in the plateau phase, as proposed by Holmgren et al [23].

The apoptotic cell death caused by such stress is of particular interest. Apoptosis during development is generally thought to be triggered by growth factors and other environmental cues [24], [25], and the role of mechanical stress in this process has only recently been considered [26], [27]. Our results suggest that inhomogeneities in the mechanical properties of the confining tissue can guide morphological changes in tumor growth, independent of cell migration, by inducing apoptosis in regions of high compressive stress and allowing proliferation in regions of low stress. Furthermore, the compression-induced apoptosis occurs via the mitochondrial pathway, a regulatory control mechanism that cancer cells with elevated Bcl-2 activity might escape to produce more malignant tumors.

## Materials and Methods

### Cell culture

Non-metastatic murine mammary carcinoma cells 67NR were obtained from the American Type Culture Collection (ATCC, Rockville, MD) and were used for most of the experiments in this study. Metastatic murine mammary carcinoma cells EMT6 were generously provide by Dr. James Freyer (Los Alamos National Laboratory, Los Alamos, NM). 67NR cells were maintained in high-glucose (4.5 mg/ml) Dulbecco's Modified Eagle's Medium (DMEM, Sigma, St. Louis, MO) supplemented with 1% non-essential amino acids (Invitrogen, Carlsbad, CA) and 10% fetal bovine serum (FBS). EMT6 cells were maintained in alpha-minimal essential medium (Mediatech, Manassas, VA) supplemented with 10% FBS. For experiments requiring glucose-free, serum-free and hypoxic environment (denoted the “starvation medium”), spheroids were cultured in glucose-free DMEM (Invitrogen) not supplemented with FBS, and in 5% CO_2_-1% O_2_-N_2_ biomedical air (Airgas East, Salem, NH).

### Anti-apoptotic transduction with Bcl-2 and crmA

One million cancer cells were seeded into a 10 cm-diameter Petri dish and transfected via addition of Lipofectamine 2000 (Invitrogen) and plasmid DNA containing the full-length murine Bcl-2 (ATCC) or cytokine response modifier A (crmA, generous gift from Dr. Brian Seed at Massachusetts General Hospital, Boston, MA) cDNA and the puromycin selectable marker. Twenty-four hours after transfection, the cells were passaged and selection medium containing 20.0 µg/ml puromycin was added. After 7–10 days of culturing in this medium, only a few colonies remained; these were pooled together and propagated in the presence of selection-level puromycin. Expression of Bcl-2 and crmA in the transfected cells was determined by quantitative real-time PCR (Bcl-2) or PCR (crmA) with appropriate primers. Once a stable transfected cell line was established, the cells were maintained in the presence of selection-level puromycin.

### PCR assays

Quantitative real-time PCR (ABI Prism 7300, Applied Biosystems, Foster City, CA) was used to determine the mRNA level of Bcl-2 gene transfected into cancer cells. The thermal cycling conditions consisted of 35 cycles of PCR amplification (denaturation: 95°C 30 sec, annealing/extension: 68°C, 1 min). The mRNA level of crmA gene was evaluated using conventional PCR assay. The following sense and antisense primers were designed using Primer2 software (Applied Biosystems): Bcl-2: 5′-GGGATGCCTTTGTGGAACTATATG and CTGAGCAGGGTCTTCAGAGACA-3′; crmA: 5′-AAGCTTATGGATATCTTCAG and GCCTGCCGCTTAATTAGTTGT-3′; and GAPDH: 5′-ACAGCCGCATCTTCTTGTGCAGTG and GGCCTTGACTGTGCCGTTGAATTT-3′.

### Culture of tumor spheroids co-embedded with micro-beads in agarose gels

To monitor spheroid growth and stress accumulation, appropriate amounts of GFP- or RFP-labeled single cancer cells, 1 µm (diameter) carboxylate-modified fluorescent beads (Molecular Probes, Eugene, OR), 2.0% (w/v) agarose (Type VII, low gelling temperature, Sigma, St. Louis, MO) stock solution (1X PBS) and cell culture medium were mixed so that the final concentrations of cells, micro-beads and agarose were 3.5×10^3^ cells/ml, 4.5×10^5^ beads/ml and 0.5% or 1.0%, respectively. The bottom of a 10 mm×2 mm (diameter×depth) cylindrical well in a 5 mm thick glass slide (custom made) was first coated with 50 µl of 1.0% cell-free agarose gel to prevent cell attachment. 300 µl of the cell/micro-beads/agarose mixture was then added to the well and allowed to polymerize for 10 minutes at room temperature. The glass slide was then placed in a 100 mm×25 mm Petri dish filled with 40 ml cell culture medium (see experimental setup in Supplementary Fig. S1*A*). The medium was replenished every 5 days.

### Imaging and quantification of matrix compression around growing spheroids

We measured the volume of growing spheroids and the 3D distribution of their surrounding micro-beads every 5–7 days using an Olympus FlouView 500 confocal microscope system (Olympus, Center Valley, PA). At each time point, a volume of 638 µm×638 µm×250 µm was imaged so that the equator of the spheroid was centered at the volume's bottom surface; the step size in Z was 1 µm. Three control image stacks of micro-beads were then acquired using the same procedure, but in nearby spheroid-free areas at least 500 µm away from any spheroid (so that the bead density was not affected by growth-induced compression). To determine the local micro-bead density as a function of distance from the spheroid, a Matlab (Mathworks, Natick, MA) routine calculated the minimum distance from each micro-bead to the spheroid surface and, subsequently, the relative density of micro-beads in 10-µm thick “shells” around the spheroid (*ρ*_beads_, normalized to the micro-bead density in the control image stacks). The strain of agarose gel in those shells is then calculated as *ε*_gel_ = 1−1/*ρ*_beads_. This procedure preserved the effect of microscopic variation in spheroid surface shape on matrix deformation.

### Culture of tumor spheroids in hanging droplets

To create tumor spheroids for the externally-applied compression experiments, we used the hanging droplet method. Briefly, 20 µl droplets of cancer cell suspension (3.0×10^5^ cells/ml for 67NR and 2.0×10^5^ cells/ml for EMT6) were added to the inside of a 10 cm-diameter Petri dish cover. After placing the cover back on the dish filled with 10 ml of culture medium, the dish was placed in the incubator (5% CO_2_, 37°C) to allow spheroid growth within each droplet. Spheroids usually reached about 300 µm in diameter after 3 days of culture.

### Compression of monolayers of cancer cells and tumor spheroids

For compression of monolayers of cancer cells, the cells were seeded on the membrane of a 6-well transwell insert with 0.4 µm pores (Corning, Lowell, MA). 1.5 ml and 2.5 ml cell culture medium were added to the upper and lower chambers of the well, respectively. After overnight incubation (for cell adhesion to the membrane), a layer of 2 mm-thick 1% agarose gel was placed on top of the cells and pistons of desired weight were then applied on the agarose gel for compression (see experimental setup in Supplementary Fig. S1*B*). During the compression, the pores in the transwell insert membrane allowed convection of fluid out of the gel and also diffusion of nutrients, growth factors and oxygen to the spheroids. For compression of tumor spheroids, the spheroids grown in hanging droplets were collected and re-suspended in 1.0% agarose solution. 1 ml of the solution was then added into a 6-well transwell insert with 0.4 µm pores in its membrane, making the gel thickness ∼2 mm. The spheroid-agarose mixture was allowed to gel for 20 min at room temperature, after which 1.5 ml and 2.5 ml cell culture medium were added to the upper and lower chambers of the well, respectively. The spheroid-gel construct was then compressed with a piston of desired weight (see experimental setup in Supplementary Fig. S1*C*).

### Staining and imaging of proliferation, caspase-3 activity and necrosis in tumor spheroids

Proliferating cells in spheroids were detected with a Cell Proliferation Fluorescence Kit (Amersham Biosciences, Buckinghamshire, UK) per the manufacture's protocol. Briefly, spheroids were labeled with a BrdU labeling reagent, fixed (1% formalin, 0.1% Triton in PBS), incubated with an anti-BrdU/Nuclease reagent and finally incubated with Cy5 labeled goat anti mouse IgG. Caspase-3 activity and necrosis in cancer cells or spheroids were detected with Nucview 488 Caspase-3 Assay Kit for live cells (Biotium, Hayward, CA) and propidium iodide (PI, Molecular Probes, Eugene, OR), respectively. Monolayers of cells were stained for 15 min and spheroid-gel constructs stained for 45 minutes at 4 °C and rinsed twice with fresh medium. The labeled cells were imaged using an Olympus FlouView 500 confocal microscope system (Olympus, Center Valley, PA). The results were quantified as the percentage of caspase-3/PI positive cells in the population.

### Immunohistochemical assays for apoptotic in tumor spheroids

Spheroids were fixed in 4% paraformaldehyde for 4 hr and then embedded in paraffin. 5 µm sections were cut from the paraffin blocks and the cancer cells were stained for apoptosis with ApopTag® (CHEMICON International, Temecula, CA). Cells were counter-stained with hematoxylin.

### Statistics

Values were characterized by arithmetic mean and standard error of the mean (SEM). Significant differences between different populations of data were tested with Student *t*-test for unpaired observations.

## Supporting Information

Supplementary Methods S1 (0.05 MB DOC)

Figure S1 Experimental setups. (A) Culturing tumor spheroids co-embedded with micro-beads in agarose gel. (B) Applying exogenous, well-defined compressive stress to a monolayer of cancer cells. (C) Applying exogenous, well-defined compressive stress to tumor spheroids grown to a desired size in hanging droplets.
(14.63 MB TIF)

Figure S2 Analysis of 2D images. (A–G) Quantifying local deformation in a tumor spheroid (green, GFP transduction) and the corresponding local strain in the agarose gel using micro-beads (red). (H–K) Quantifying local fraction of necrotic area (red, propidium iodide staining) in a spheroid (green, GFP transduction). (L) Correlating the asymmetries in spheroid shape and in gel strain. (M) Correlating the asymmetries in spheroid necrosis and in gel strain.
(5.25 MB TIF)

Figure S3 The development of caspase-3 activity (green, left column) and necrosis (red, central column) in growing tumor spheroids (transmitted image superimposed with the caspase-3 and necrosis images, right column) in 1% agarose. Scale bar = 20 µm.
(7.48 MB TIF)

Movie S1 3D rendering of an oblate tumor spheroid cultured in 0.5% agarose gel.
(3.13 MB MOV)

Movie S2 Image analysis to quantify local tumor spheroid deformation
(0.45 MB MOV)

Movie S3 Image analysis to quantify local strain in agarose gel caused by tumor spheroid growth.
(0.56 MB MOV)

Movie S4 Image analysis to quantify local cell death in tumor spheroid.
(0.31 MB MOV)
