# Supplementary material for: Micro-Environmental Mechanical Stress Controls Tumor Spheroid Size and Morphology by Suppressing Proliferation and Inducing Apoptosis in Cancer Cells
Source: PLoS One. 2009 Feb 27;4(2):e4632. doi: 10.1371/journal.pone.0004632 (PMC2645686; doi:10.1371/journal.pone.0004632)
Supplement: Supplementary Methods S1 — (0.05 MB DOC) [file pone.0004632.s001.doc]

# supplementary methods

### Correlation of spheroid shape with mechanical stress distribution

To correlate spheroid shape with mechanical stress distribution, 2D images of spheroids and the surrounding micro-beads were acquired at the spheroids’ equators. The images were then processed with the following procedure (Fig. S2*A*-2*G* and 2*L*):

1. The spheroid image was first thresholded and despeckled (Fig. S2*B*). Next, the perimeter of the spheroid was segmented using the following procedure: the longest axis between any two points on the perimeter was determined (blue dashed line in Fig. S2*C*). Lines ~10 μm apart were then drawn perpendicular to this axis. The points of intersection between these perpendicular lines and the spheroid perimeter were used to define the “chords” for the analysis. Bands extending perpendicularly to these chords were then drawn (sides of bands are marked with pairs of white, magenta and cyan lines in Fig. S2*C*). To quantify how much the spheroid was deformed within each band, a circle (white dashed line in Fig. S2*C*) was drawn whose diameter was the longest axis of the spheroid and the deformation index for each band was then calculated as (Fig. S2*D*)

For a demonstration of the above image analysis process, see Movie S2.

1. The micro-bead image was thresholded (Fig. S2*E*) and the shortest distance from each micro-bead pixel to the spheroid perimeter was calculated (Fig. S2*F*; intensity of red is proportional to the shortest distance to spheroid perimeter, which is outlined with the green line). The spheroid perimeter was then dilated to create a 10 μm-thick shell that overlapped with the bands constructed in Step (1), creating shell segments. The local strain in the first 10-μm thick shell of agarose gel is then calculated as (Fig. S2*G*):

For a demonstration of the above image analysis process, see Movie S3.

1. The values of *λ*_sphd,local_ and *ε*_gel,1,local_ were then averaged for the two halves of the spheroid divided by the longest axis.
2. To correlate the asymmetries in spheroid shape and in gel strain for many spheroids, we calculated “Spheroid Shape Asymmetry” and “Gel Strain Asymmetry” as shown in Fig. S2*L*, where *L_maj_* and *L_min_* are the length of the major and minor axes, respectively; *ε*_maj,i_ and *ε*_min,i_ (i = 1, 2, 3, and 4) are the strain in agarose gel in the corresponding segments along the major and minor axes, respectively.

### Correlation of cell death with mechanical stress distribution

To correlate cell death within spheroids to mechanical stress distribution, 2D images of spheroids, necrotic cells inside and the surrounding micro-beads were taken at the spheroid equators. The images were then processed with the following procedure (Fig. S2*H*-2*K* and 2*M*):

1. The spheroid image was thresholded and segmented as described in “Correlation of spheroid shape with mechanical stress distribution”. The necrosis image was then thresholded (Fig. S2*H*-2*I*) and the local necrotic fraction within each band of the spheroid calculated as (Fig. S2*K*):

For a demonstration of the above image analysis process, see Movie S4.

1. The micro-bead image was processed and the local strain in agarose gel (*ε*_gel,1,local_) calculated as described in “Correlation of spheroid shape with mechanical stress distribution”.
2. The values of δ_sphd,local_ and ε_gel,1,local_ were then averaged for the two halves of the spheroid divided by the longest axis in the spheroid.
3. To correlate the asymmetries in spheroid necrosis and in gel strain for many spheroids, we calculated “Gel Strain Asymmetry” as shown in Fig. S2*L* and “Spheroid Necrosis Asymmetry” as shown in Fig. S2*M*, where δ_maj,i_ and δ_min,i_ (i = 1, 2, 3, and 4) are the necrotic fraction in the corresponding spheroid segments along the major and minor axes, respectively.
