# Supplementary figures and images for: Micro-Environmental Mechanical Stress Controls Tumor Spheroid Size and Morphology by Suppressing Proliferation and Inducing Apoptosis in Cancer Cells
Source: PLoS One. 2009 Feb 27;4(2):e4632. doi: 10.1371/journal.pone.0004632 (PMC2645686; doi:10.1371/journal.pone.0004632)

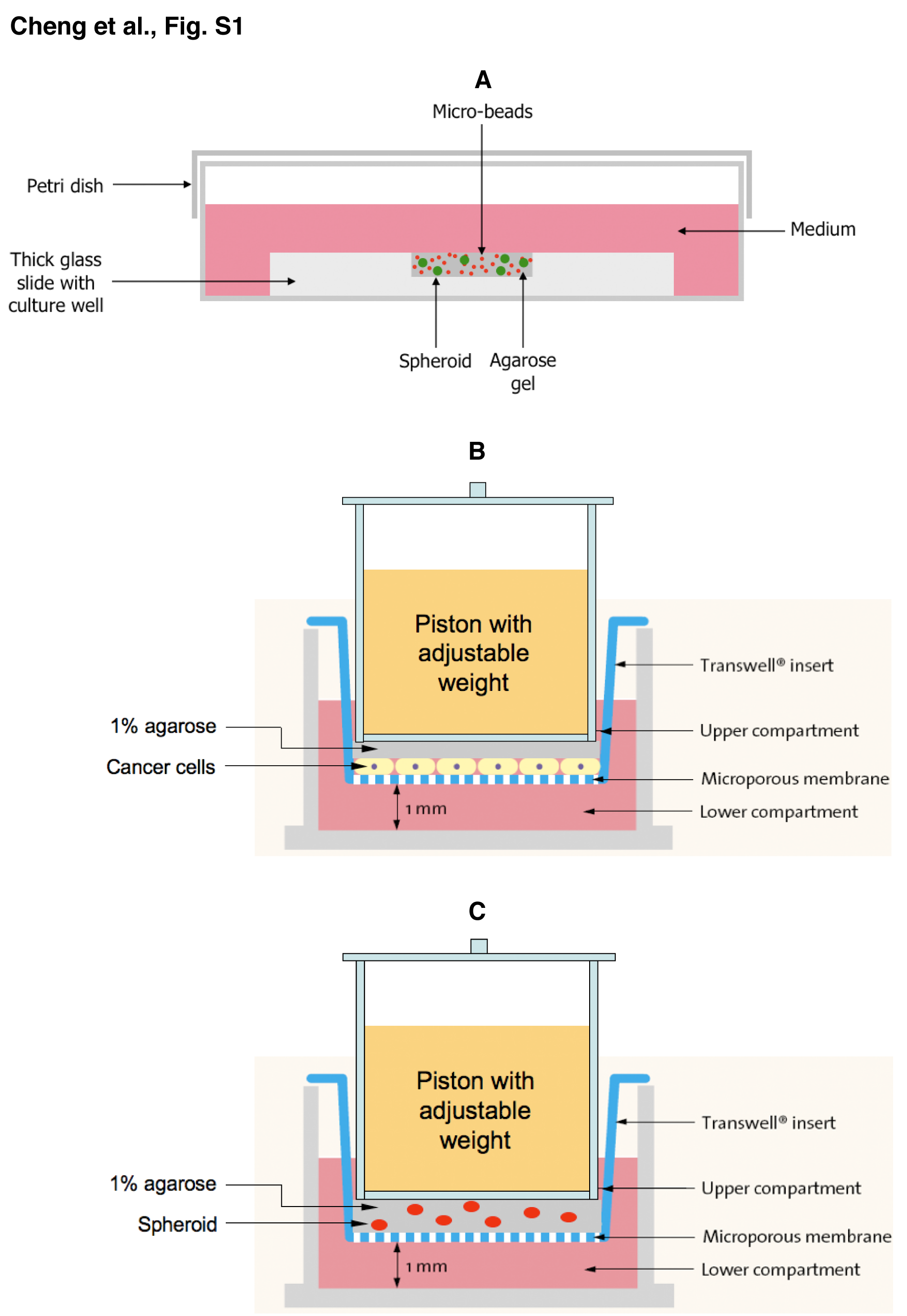

Supplement: Figure S1 — Experimental setups. (A) Culturing tumor spheroids co-embedded with micro-beads in agarose gel. (B) Applying exogenous, well-defined compressive stress to a monolayer of cancer cells. (C) Applying exogenous, well-defined compressive stress to tumor spheroids grown to a desired size in hanging droplets. (14.63 MB TIF) [file pone.0004632.s002.tif]

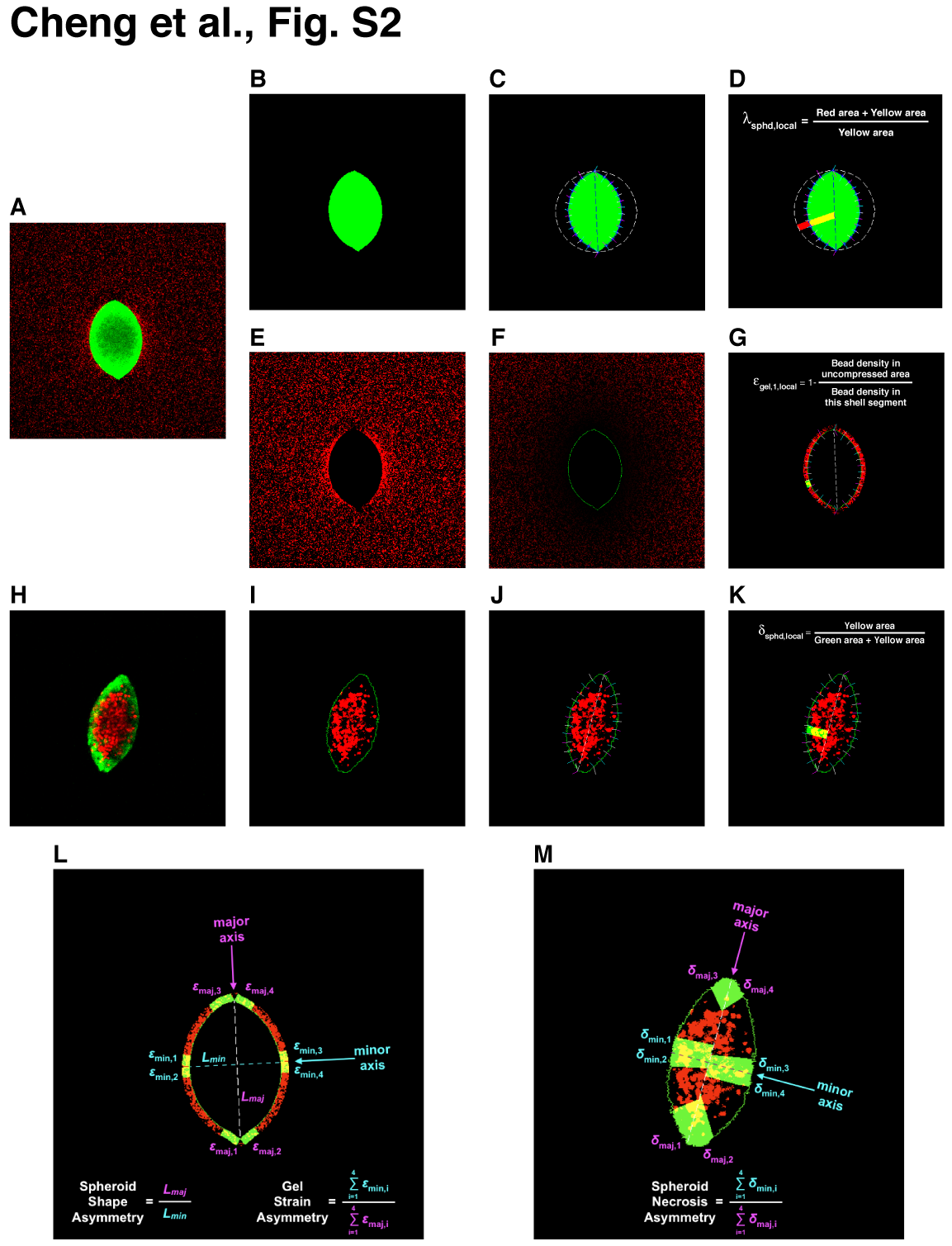

Supplement: Figure S2 — Analysis of 2D images. (A–G) Quantifying local deformation in a tumor spheroid (green, GFP transduction) and the corresponding local strain in the agarose gel using micro-beads (red). (H–K) Quantifying local fraction of necrotic area (red, propidium iodide staining) in a spheroid (green, GFP transduction). (L) Correlating the asymmetries in spheroid shape and in gel strain. (M) Correlating the asymmetries in spheroid necrosis and in gel strain. (5.25 MB TIF) [file pone.0004632.s003.tif]

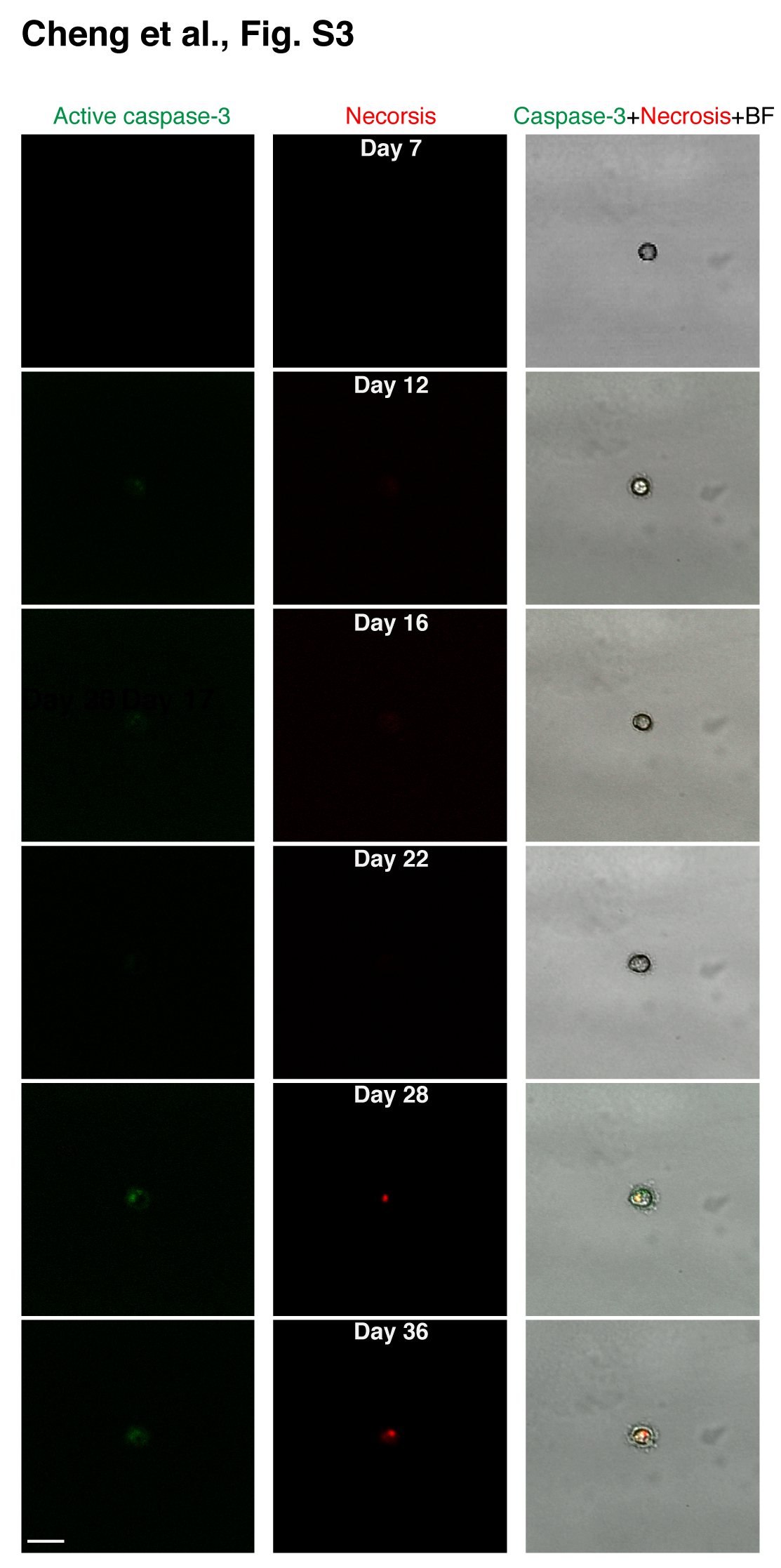

Supplement: Figure S3 — The development of caspase-3 activity (green, left column) and necrosis (red, central column) in growing tumor spheroids (transmitted image superimposed with the caspase-3 and necrosis images, right column) in 1% agarose. Scale bar = 20 µm. (7.48 MB TIF) [file pone.0004632.s004.tif]
